# Sustainable implementation of a frailty education program for formal health care providers

**DOI:** 10.3389/fpubh.2025.1654098

**Published:** 2025-11-12

**Authors:** Matthew J. Sargent, Margaret Chen-Mei Lin, Grace Park, Shannon Parsons, Shannon Freeman

**Affiliations:** 1 Centre for Technology Adoption for Aging in the North, University of Northern British Columbia, Prince George, BC, Canada; 2 Fraser Health Authority, Surrey, BC, Canada; 3 School of Nursing, University of Northern British Columbia, Prince George, BC, Canada

**Keywords:** frailty, education, educational module, eLearning, aging, frailty prevention, evaluation

## Abstract

**Background:**

Frailty is a significant contributor to morbidity and mortality and places considerable strain on healthcare systems. Frailty education is essential for shaping professional attitudes and enabling proactive care. The Canadian Frailty Network’s AVOID (activity, vaccination, optimization of medications, interactivity, diet) framework was released in 2019 to help prevent and mitigate frailty. An interdisciplinary team of health system leaders, clinicians, and academics adapted the AVOID framework into an educational module for healthcare providers. This study evaluated the effectiveness of the module and provides recommendations for developers of eLearning modules.

**Materials and methods:**

This study employed a convergent mixed-methods design. Participants included a diverse sample of healthcare providers from a Canadian health authority, including nurse educators, physiotherapists, and care aides, who completed the AVOID Frailty educational module through an online learning platform. Participants completed surveys before and after completing the module, probing their understanding of frailty management and perspectives on the module. A subsample of individuals who completed the module participated in one of four focus groups with the evaluation team. Quantitative survey data were analyzed descriptively, and qualitative focus group and survey data underwent an exploratory descriptive analysis led by two members of the evaluation team. Data were integrated during analysis where appropriate.

**Results:**

The module improved participants’ self-reported knowledge of frailty assessment, mitigation, and prevention. Participants valued the module’s length and content but identified a need for more interactive and visually engaging elements, as well as clearer guidance on practical implementation. Participants intended to use resources from the module, but noted that limitations of resources in the healthcare system could pose challenges for frailty prevention initiatives.

**Conclusion:**

This study suggests areas for improvement of the AVOID Frailty educational module, highlighting the importance of including healthcare staff perspectives when developing eLearning modules. Further, this work underscores the potential of targeted education to strengthen frailty care.

## 1 Introduction

Frailty is a state of decreased resilience to stressors, which can be identified through phenotypic characteristics including unintentional weight loss, exhaustion, weakened strength, slow walking speed, and low physical activity (1). Health care systems face great economic costs for sustaining frail individuals (2). In 2022, 47% of health care expenditure in Canada was spent on people over 65 years of age, despite this group accounting for only 19% of the population (3). Frailty is often associated with aging; however, aging does not necessitate the development of frailty (4). Interventions can mitigate or prevent frailty (5). Education around frailty is important for managing and preventing frailty (6). Viggars et al. (6) suggest that frailty education is beneficial for care providers, health care professionals, and older adults. Indeed, frailty presents challenges for older adults, their care providers, and health care systems. However, frailty can be prevented and mitigated, and education is a critical component of frailty management. A systematic review on frailty education programs for health care professionals found that frailty assessment and management are infrequently incorporated into care practice, and that training materials on frailty are often not structurally evaluated or reported (7). There are also even less health care provider education materials that focus solely on frailty.

The Pacific Regional Centre for Healthy Aging (PRCHA) implements large-scale projects to promote healthy aging in British Columbia (BC), Canada. Part of PRCHAs’ initiative to promote healthy aging involved an eLearning (i.e., delivered digitally through the internet) frailty educational module, the development of which was led by Fraser Health Authority (FHA). The development of this module was spearheaded by a clinical nurse educator with a home and community health team from FHA, accompanied with regular consultation with several representatives from different regional health authorities. The module, termed the AVOID Frailty educational module, was informed by the AVOID (Activity, Vaccination, Optimization of Medications, Interaction, and Diet and Nutrition) framework (8). This framework emphasizes the importance of these factors in successful frailty prevention and management. Two versions of the AVOID Frailty educational module were developed; one version, which contains information on frailty prevention and mitigation, is intended for community health workers and health care aides. The other module was designed for all other applicable health professionals. This module contains the same information as the module for community health workers and health care aides, with additional education about frailty assessment. Throughout this paper, references to the AVOID Frailty educational module will refer to both versions of the module, while discussion of frailty assessment will be based exclusively on the version of the module which contains that information.

The AVOID Frailty educational module is a self-directed online learning module structured around the AVOID framework, which was originally released by the Canadian Frailty Network in 2019 to help prevent and minimize frailty (9). The module is hosted on an online learning platform used by several health authorities in BC, called LearningHub, where health authority staff can log in to the platform and enroll in courses or education modules. The AVOID Frailty educational module is composed of several sections: Definition of Frailty, Frailty Assessment, Frailty Mitigation using the AVOID Strategy, Frailty Pathway, and Resources. In the Definition of Frailty section, content focuses on how to identify the consequences and risk factors of frailty. In the Frailty Assessment section, content focuses on three tools: the Frailty Index (10), Clinical Frailty Scale (CFS) (11), and Comprehensive Geriatric Assessment (CGA) (12). After learning about the three tools, there are also interactive activities (one matching test and two case studies) that test the learners’ understanding of the CFS. The third section, Frailty Mitigation using the AVOID Strategy, shares frailty mitigation and prevention ideas informed by the AVOID Frailty framework. The Frailty Pathway section shares information about FHA’s Frailty Pathway, which is a model intended to help care providers assess older adults’ level of frailty using the CFS and care plan with tools like the CGA, AVOID Frailty Framework, and FHA’s Frailty Pathway resource guide to manage frailty with older adults. In the Resources section of the module, a tab lists all the resources shared in the module to support care providers in finding resources more easily in the future.

The module was designed to be completed in one session, which lasted approximately 20 min for most participants. As the module was delivered online, participants could log in and complete the module when it was convenient for them. The module development team collaborated with community health leads and administrators to share the module with frontline care providers. The team also shared the module with representatives from different regional health authorities and encouraged each health authority to consider strategies that could support the education uptake.

This paper describes the evaluation process and outcomes of the AVOID Frailty educational module, which was intended to contribute to better understanding and improvement of frailty education for formal health care providers. Findings from the evaluation were used to refine the module to optimize completion, engagement, and learning. These findings provide broad recommendations for sustainable implementation, or effective long-term uptake, of eLearning educational modules for formal health care providers. Sustainable implementation of eLearning modules can be achieved in cases where the completion of eLearning modules is integrated as part of regular training for health care staff, and the eLearning modules have been refined using feedback from end-users to improve modules while maintaining their strengths. The aim of this study was to provide a comprehensive evaluation of the AVOID Frailty educational module for FHA’s quality improvement and extrapolate these insights to provide broad recommendations for the sustainable implementation of eLearning educational modules targeting formal health care providers.

## 2 Materials and methods

### 2.1 Evaluation objectives

The objectives of our evaluation of the AVOID Frailty educational module were:

1. Detail participants, and their perspectives on utility of information from the AVOID Frailty educational module and perceived potential benefits to patients following completion of the module.
2. Measure changes in participants’ understanding of frailty management (assessment, mitigation, and prevention) after completion of the educational module.
3. Describe participant experiences completing the AVOID Frailty educational module and assess feasibility of incorporating module strategies into clinical practice.

### 2.2 Evaluation approach

This evaluation employed a convergent mixed-methods approach where data collected from surveys and focus groups were analyzed to evaluate the AVOID Frailty educational module. Survey data were collected from the LearningHub database by a clinical nurse educator. Focus groups were facilitated by the evaluation team.

#### 2.2.1 Surveys

The LearningHub platform, where the module was delivered, allows module developers to include surveys along with educational content. Survey questions were formulated as part of the evaluation. All participants who registered for the AVOID Frailty educational module and started the module were presented with a short survey before they viewed any content from the module (pre-module survey). Additionally, participants were presented with another survey after they completed the module (post-module survey). The surveys were not mandatory, and participants could choose to skip or not complete the survey if they preferred. Data were collected by a clinical nurse educator from FHA through LearningHub and delivered to the evaluation team in de-identified format. The pre- and post- module surveys are available in the Supplementary material.

As all participants who registered for the AVOID Frailty educational module were presented with the opportunity to complete the pre-module and post-module surveys, no explicit inclusion or exclusion criteria were defined. The analysis in this study used all survey data from FHA staff who registered for the AVOID Frailty educational module and chose to complete the pre-module and post-module surveys. Numerical survey data were analyzed to present descriptive statistics about module reach and engagement and changes in understanding of frailty assessment, mitigation, and prevention after module completion. Open-ended data from surveys underwent a descriptive analysis to explore trends in participant responses and to identify quotes to illustrate participant feedback on the module. Two evaluation team members read over the responses to open-ended survey questions to identify trends across participant responses and select illustrative quotes.

#### 2.2.2 Focus groups

To better understand user experiences and enhance evaluation, four semi-structured focus groups were conducted to discuss issues related to the AVOID Frailty educational module. The clinical nurse educator and colleagues in the regional home and community overseeing this project recruited focus group participants via email and verbal invitation. The email included both promotional messages inviting staff to learn about frailty management through the module, and recruitment messages inviting staff who completed the module to join a focus group. The email was sent to home health office and assisted living team managers, and the managers helped distribute the email to their team’s care providers. The clinical nurse educator also shared information about the module in the managers’ routine regional meetings. To participate in focus groups, prospective participants must have completed the AVOID Frailty educational module and contacted the clinical nurse educator to express their interest in participating in a focus group which was to be held over Zoom. Focus group scripts were designed by the evaluation team and are available in the Supplementary material.

Qualitative data from focus groups were analyzed descriptively to explore trends in participants’ perspectives on the AVOID Frailty educational module. Two members of the evaluation team read the focus group transcripts in their entirety to identify trends in responses within and across groups. The two evaluation team members involved in the focus group analysis discussed points of interest and worked together to resolve disagreements and identify quotes which could be used to illustrate participant feedback. The data underwent an exploratory, descriptive analysis rather than a traditional thematic analysis. Because this evaluation stemmed from a quality improvement initiative for FHA, the transcripts did not undergo line-by-line coding, and the analysis was not formally assessed for inter-rater reliability. Further, while many speakers in the focus groups were identified, it was not possible to identify all speakers as only audio recordings were maintained and transcribed. Due to our exploratory analysis strategy, the weight given to certain perspectives may not be directly reflective of the length of time spent discussing a perspective or the number of participants endorsing a perspective. While this approach is less exhaustive than a traditional thematic analysis, it was deemed appropriate for identifying high-level perspectives on the AVOID Frailty educational module which could be used by FHA for improvement of the module.

### 2.3 Ethical considerations

Data collection was facilitated by SP as part of a quality improvement initiative for FHA, thus Research Ethics Board review was not required, and the study was not reviewed by the local research ethics board. Participants were informed prior to focus groups that the focus groups would be recorded and that de-identified quotes may be used in presentations, reports, and manuscripts. Participants were also told that participation was voluntary and that they were free to choose not to answer any questions. They were also informed that they could choose to withdraw their responses even after their data had been collected. Program evaluation activities and quality improvement studies are exempt from Research Ethics Board review as stated by the Tri-Council Policy Statement: Ethical Conduct for Research Involving Humans (13).

## 3 Results

Quantitative and qualitative data were used to assess the objectives of this evaluation. Quantitative data were used to evaluate changes in participants’ understanding of frailty management (frailty assessment, mitigation, and prevention), while qualitative survey data were incorporated to provide more details on the types of learnings participants may have gained from the module or criticisms participants may have had about their learning experience. Quantitative data were also used to assess whether participants intended to use resources from the module in their practice. Qualitative data, from focus groups and post-module surveys, were used to gather more fulsome insights on which resources from the module clinicians intended to use in their practice, the feasibility of incorporating strategies from the module into clinical practice, benefits to patients that may arise due to staff participation in the module, and overall strengths and limitations of the AVOID Frailty educational module. Data were integrated during the analysis where appropriate to best answer the evaluation objectives. Specifically, quantitative data were used to provide high-level descriptive statistics while qualitative data were used to provide more detailed, fulsome insights.

In the first three months in which the AVOID Frailty educational module was offered to formal care providers in one health region (FHA), 1,509 participants had completed the module and responded to the pre-module survey. Detailed information about the participant sample who responded to the pre-module survey in both modules is presented in Table 1. A description of the roles of focus group participants is displayed in Table 2.

**Table 1:** Professional roles of pre-module survey participants in AVOID Frailty educational module.

Professional role	Number of module participants (Nurse/Allied health) (*N* = 327)	Number of module participants (Community health worker/Health care aide) (*N* = 1,182)
Academic	2 (0.6%)	1 (0.1%)
Nurse	189 (57.8%)	81 (6.9%)
Allied health professional	81 (24.8%)	182 (15.4%)
Other	29 (8.9%)	477 (40.4%)
Community member	14 (4.3%)	284 (24.0%)
Unregulated health professional	5 (1.5%)	111 (9.4%)
Leadership/corporate	3 (0.9%)	2 (0.2%)
Administrator	2 (0.6%)	0 (0.0%)
Physician	1 (0.6%)	2 (0.2%)
Student	1 (0.3%)	34 (2.9%)
Nurse practitioner	0 (0.0%)	8 (0.7%)

**Table 2:** Professional roles of participants in focus groups for the AVOID frailty educational module.

Focus group 1: Nurses	Focus group 2: Allied health professionals	Focus group 3: Managers	Focus group 4: Care aides/Community health workers
Clinical nurse educators (*n* = 2) Clinical practice leads (*n* = 2) Clinical research nurse (*n* = 1) Registered nurses (*n* = 2)	Occupational therapists (*n* = 3) Physiotherapist (*n* = 1) Caregiver support clinician (*n* = 1)	Regional manager (*n* = 1) Team lead (*n* = 1) Regional team lead (*n* = 1)	Community support workers (*n* = 4)

**Figure 1:**
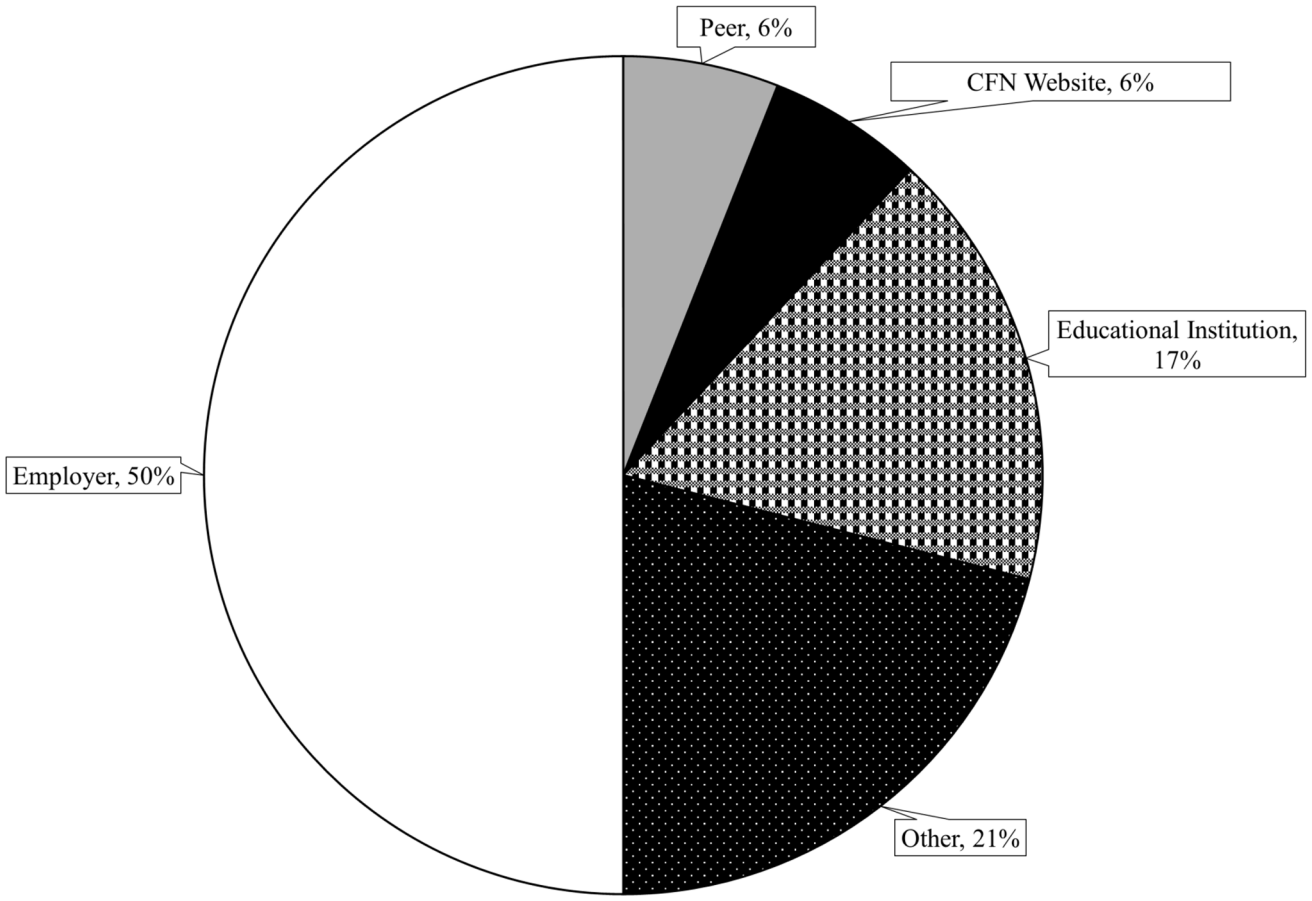
Methods by which survey participants learned about the AVOID framework.

Participants learned about the AVOID Frailty educational module through different means. Twenty-four percent of participants reported having previously heard of the AVOID framework before completing the educational module (*n* = 359). Of these, participants reported hearing about it from their employer (50%), an educational institution (17%), a peer (6%), the Canadian Frailty Network website (6%), and other sources (21%). Methods by which participants learned about the AVOID framework are displayed in Figure 1.

A focus group participant mentioned that they learned about the AVOID Frailty educational module through a regional geriatric convention before completing the module on LearningHub:

“A couple of months ago I went to an in-person kind of geriatric convention that they had … they actually had brought up the whole AVOID and they actually brought everything up that I went through after when I went into the [online site], all the same slides and stuff, so yeah we knew it's been coming and I did see the slides previous and then again through [clinical resource nurse] and the employer.” (*Participant 1, Focus Group 1, Clinical Practice Lead*).

Another participant described learning about the AVOID Frailty educational module from their peers in a home health home support weekly connection meeting:

“[It] was one of the monthly meetings where I had first heard [clinical resource nurse] and [PRCHA team member] talk about it. And then we do have home health home support weekly connections where [clinical resource nurse, community manager], myself, and one of the regional educators for home support connect weekly just to see what's been going on, what's in the works, where is the collaboration needed. We work collaboratively on a lot of stuff. So that's where [clinical resource nurse] had also let us know that this is coming. We were well informed about the go live time implementation education once they were beta-testing, we were involved in that as well. So we had a few community health workers who participated in beta-testing. So it was all wonderful communication we had from [clinical resource nurse].” (*Participant 2, Focus Group 3, Team lead*).

### 3.1 Changes in understanding of frailty assessment

**Figure 2:**
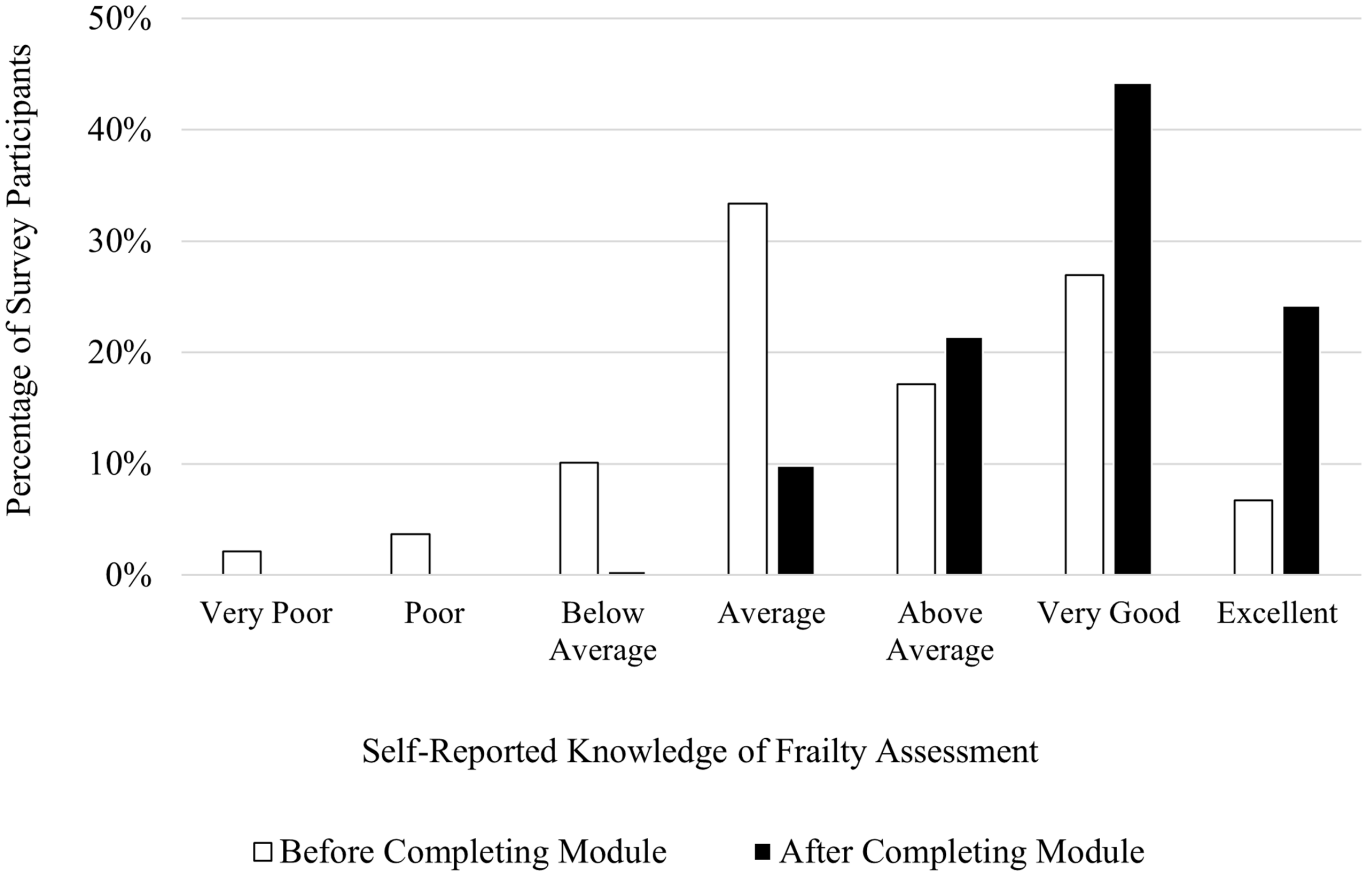
Participants’ self-reported knowledge of frailty assessment before (*N* = 327) and after (*N* = 285) completing the AVOID Frailty Educational Module. *χ*^2^ (6*, N* = 612) = 126.00, *p* < 0.001.

Participants reported that their understanding of frailty assessment increased upon completion of the AVOID Frailty educational module. Approximately one third of participants rated their knowledge of frailty assessment as “Excellent” or “Very Good” before completing the module (34%), while 68% of participants rated their knowledge of frailty assessment as “Excellent” or “Very Good” upon completion of the module. Six percent of participants rated their knowledge of frailty assessment as either “Poor” or “Very Poor” prior to completing the module, while after completing the module, 0% of participants rated their knowledge of frailty assessment as either “Poor” or “Very Poor.” A chi-square test for homogeneity revealed a significant difference in the distribution of knowledge levels of frailty assessment for participant samples before and after completing the module, *χ*^2^ (6, *N* = 612) = 126.00, *p* < 0.001. Figure 2 shows participants’ ratings of their knowledge of frailty assessment before and after completing the AVOID Frailty educational module. Qualitative insights from survey data supported that participants’ knowledge of frailty assessment had increased upon completion of the educational module. For instance, in response to the post-survey question “What are two of your key takeaways from the AVOID-based educational module?,” one participant who completed the post-module survey wrote “*I know more about frailty and the clinical frailty scale that helped my knowledge expand*.” In response to the same question, another participant wrote, “*[P]ointed out tools such as frailty Index, AVOID frailty screening tool, and [electronic Comprehensive Geriatric Assessment] to identify frailty which was new to me*.”

### 3.2 Changes in understanding of frailty mitigation

**Figure 3:**
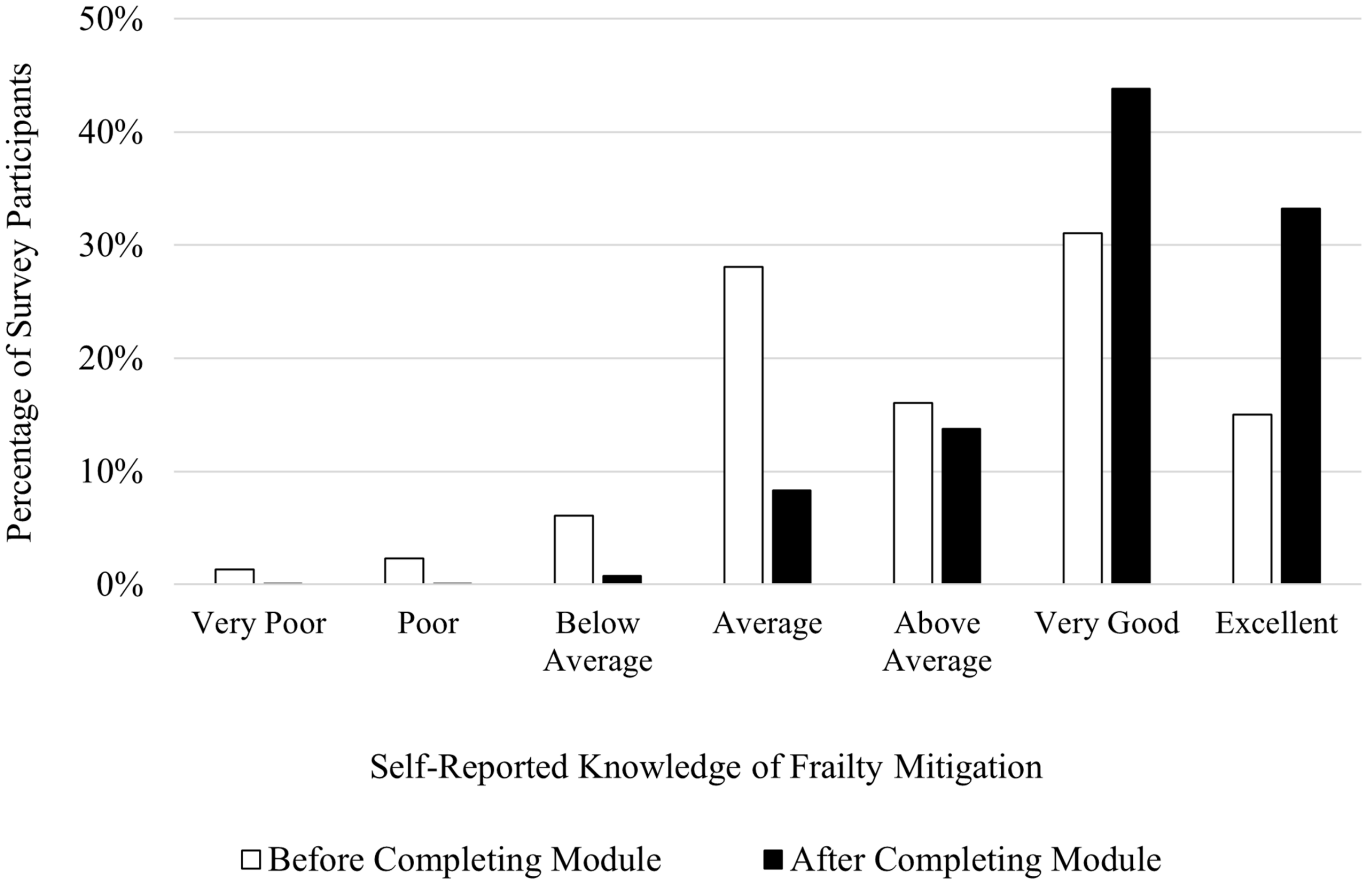
Participants’ self-reported knowledge of frailty mitigation before (*N* = 1,509) and after (*N* = 1,433) completing the AVOID Frailty educational module. *χ*^2^ (6, *N* = 2,942) = 398.47, *p* < 0.001.

Participants reported that their understanding of frailty mitigation increased upon completion of the AVOID Frailty educational module. Forty-six percent of participants rated their knowledge of frailty mitigation as “Excellent” or “Very Good” before completing the module, in contrast to 73% of participants rating their knowledge of frailty mitigation as “Excellent” or “Very Good” upon completion of the module. Four percent of participants rated their knowledge of frailty mitigation as either “Poor” or “Very Poor” prior to completing the module, while after completing the module, < 1% of participants rated their knowledge of frailty mitigation as either “Poor” or “Very Poor.” A chi-square test for homogeneity revealed a significant difference in the distribution of knowledge levels of frailty mitigation for participant samples before and after completing the module, *χ*^2^ (6, *N* = 2,942) = 398.47, *p* < 0.001. Figure 3 shows participants’ ratings of their knowledge of frailty mitigation before and after completing the AVOID Frailty educational module. Additionally, insights from survey data suggested that participants gained an increasing awareness of strategies for frailty mitigation upon completion of the module. One participant who completed the post-module survey described two of their key takeaways, writing “*1. Frailty is not normal part of aging. 2. Utilization of AVOID framework can prevent and delay progression of frailty.*” Another participant who completed the post-module survey wrote that two of their key takeaways were, “*There are several non medical interventions that impact someone’s level of frailty and early identification/prevention can make a big difference for older adults quality of life.*”

### 3.3 Changes in understanding of frailty prevention

**Figure 4:**
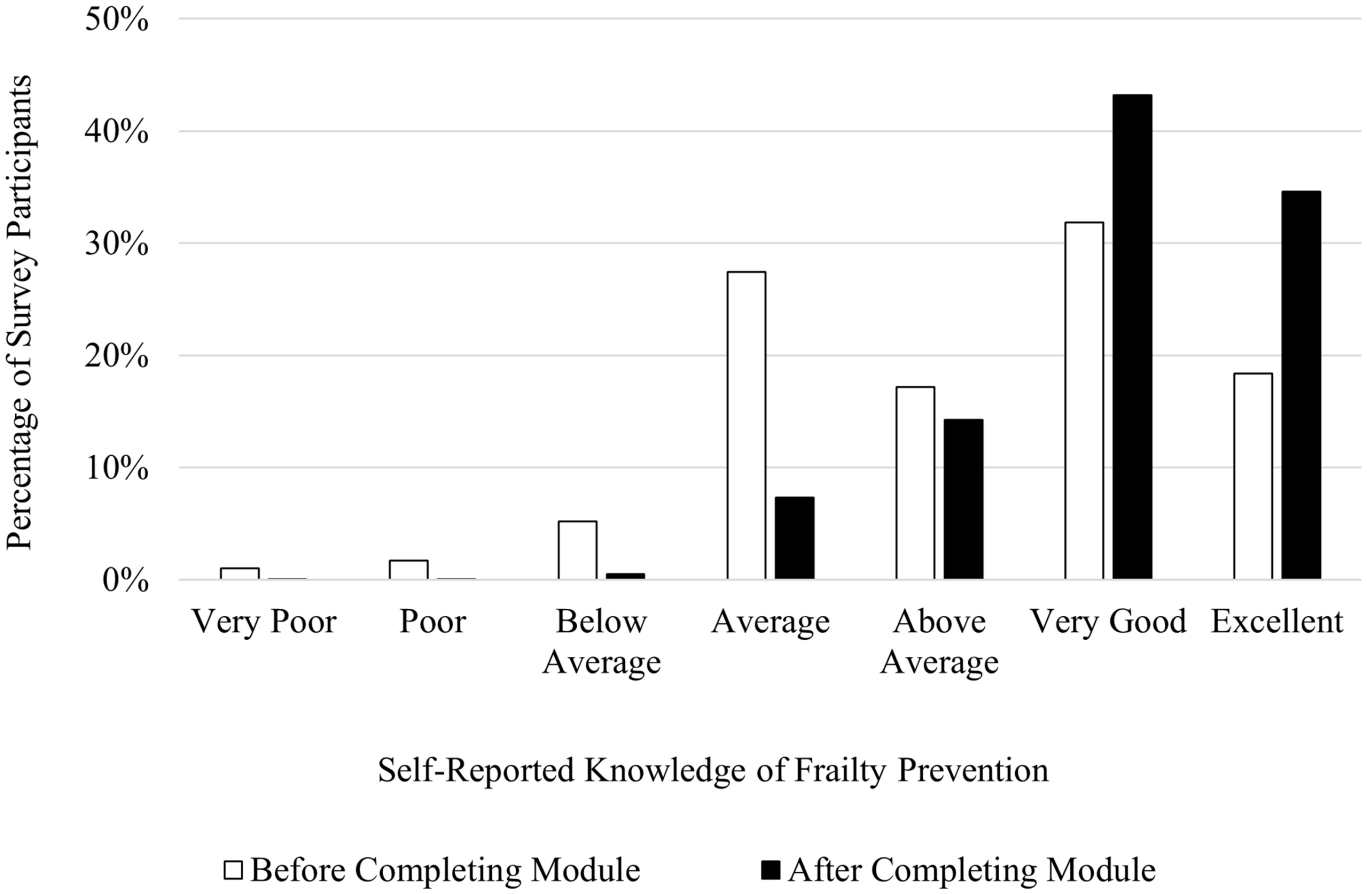
Participants’ self-reported knowledge of frailty prevention before (*N* = 1,509) and after (*N* = 1,433) completing the AVOID Frailty educational module. *χ^2^* (6, *N* = 2,942) = 357.56, *p* < 0.001.

Participants reported that their understanding of frailty prevention increased upon completion of the AVOID Frailty educational module. Forty-nine percent of participants rated their knowledge of frailty prevention as “Excellent” or “Very Good” before completing the module, while 78% of participants rated their knowledge of frailty prevention as “Excellent” or “Very Good” upon completion of the module. Three percent of participants rated their knowledge of frailty prevention as either “Poor” or “Very Poor” prior to completing the module, while after completing the module, < 1% of participants rated their knowledge of frailty prevention as either “Poor” or “Very Poor.” A chi-square test for homogeneity revealed a significant difference in the distribution of knowledge levels of frailty prevention for participant samples before and after completing the module, *χ*^2^ (6, *N* = 2,942) = 357.56, *p* < 0.001. Figure 4 shows participants’ ratings of their knowledge of frailty prevention before and after completing the AVOID Frailty educational module. Participants who completed the post-module survey also described key takeaways from the module which demonstrated an improved understanding of frailty prevention. For instance, in response to the survey question “What are two of your key takeaways from the AVOID-based educational module?” a survey participant wrote, “*Frailty is not a normal part of aging; early identification to support intervention to promote healthy aging*.” However, while most participants felt the module helped them learn more about frailty prevention, not all participants felt the module met their learning goals. In response to the post-survey question, “What were your learning goals, and were they met? Please describe,” one participant wrote “*How to promote decreased frailty/ not really met as there is limited ability for me to do more than I am already doing to make a difference.*”

### 3.4 Utility of resources in the AVOID educational module

Participants desired to incorporate resources from the AVOID Frailty educational module into their practice. Most participants who completed the post-module survey stated that they would be using resources from the module in the future. Resources participants intended on using after module completion included the Canadian Frailty Network website (14) Healthy Eating for Seniors Guide (15), Comprehensive Geriatric Assessment (12), Five Questions to Ask About Your Medications (16), and Active Aging Canada (17). A participant indicated that they would have found it useful if they could directly access resources from the module after the module was completed, rather than needing to take note of the resources while completing the module:

“There was two [screening tools] in the education module. If you looked at, you could click on resources as you went through it. And I should have noted them down. And, at the end during the feedback portion. If there was, all those resources there that I could click on. I felt like I wanted to click on them otherwise I'd have to go back into the module and, kinda find it again but I wasn't sure it would kick me out or I'd lose my spot.” *(Participant 1, Focus Group 1, Clinical practice lead).*

Overall, participants intended to use a variety of resources which were available in the module and were interested in further accessing resources upon completion of the module.

### 3.5 Feasibility of incorporating module strategies into clinical practice

Participants appreciated the educational module content, noting that it succinctly summarized information they had previously learned or that they obtained new resources for frailty management through completing the module. Some staff discussed barriers to implementing strategies from the module into their practice. A participant discussed the challenges of working within a service model which is focused on mitigation rather than prevention, noting that the amount of time they spend working with patients to mitigate frailty makes it difficult to find time to effectively work with patients to prevent frailty:

“What is really sad to me is the barriers that are associated with a lot of these tools with the inflation, cost of living, fixed income, regardless if you have a pension. Accessing the appropriate resources or having the resources available to tackle this when you do see an issue.” *(Participant 2, Focus Group 1, Clinical Nurse Educator).*

Participants identified that limitations to staff time and resources could prevent strategies from the AVOID Frailty educational module from being fully integrated into practice. A participant explained that formal care providers are likely already aware of much of the information from the module, and already practice frailty management in their day-to-day work, but noted that the module provides a holistic lens for frailty management:

“It's just bringing that thinking … pulling it together and realizing it's all interconnected at the end of the day and it's not separate systems that we're worried about. It's about the whole picture, right…. [W]e are very proactive with getting activity in the homes and things like that. So I don't see it being like, oh, an additional, oh my goodness, this is something else we have to do. It's just, bringing it into one kind of like, okay, let me just think about it as a whole, right?” *(Participant 1, Focus Group 3, Community Health Services Manager).*

When asked to speak to the feasibility of incorporating strategies from the module into their practice, formal care provider participants appreciated the holistic model of frailty management detailed by the AVOID framework. However, multiple barriers to implementation were identified, including financial concerns, a lack of staff time and resources, and a care model which focuses on frailty mitigation rather than prevention. Another limitation discussed by participants who completed the module was an apparent shortage of tangible steps which formal care providers could take to put the background information on frailty from the AVOID Frailty educational module into practice. One participant who completed the post-module survey wrote:

“This module is great as an awareness campaign for people who don’t already know that activity, vaccination, optimizing meds, diet/nutrition, and social connection have significant impacts on the health of all individuals. What it lacks is what to do about those things. What should frontline staff be doing to assess and address these factors? The module doesn’t provide any direction to the learner on how to put the information into practice; there is no direction in terms of tangible action. Other than mentioning referring to social prescribing, with no direction on how one would do that, there is no follow up actions specific to the prevention/mitigation factors.” *(Survey Participant 1).*

A related sentiment was expressed by a formal care provider who shared concerns about how to effectively use the information in the module to support clients:

“[The module] was great for the clinician … review or learning or whatever, but I thought where it missed some points was, how the clinician can provide that better care for the client. It would have been nice to be like, for the clinician: Here are some resources on how you can support those clients. For instance, did you know about the CVC program who can track the clients who are … on that edge? Did you know about the blue senior’s book? To hand out to clients. Like I felt there was not enough resources for the clinicians ourselves on, great. It's great. I have that assessment. What do I do with that assessment now? How do I support these clients? Because at the end of the day, it doesn't matter how great our assessments are if it's not getting to that client, like for instance if they go to seniors like they go to rec centers should we have a booklet in the rec centers or whatever, to prevent that frailty, because that's what people are trying to do.” *(Participant 3, Focus Group 1, Registered Nurse).*

### 3.6 Benefits to patients from staff participation in the AVOID educational module

Benefits to patients were discussed by formal care providers who participated in focus groups. A participant discussed how empowering patients with knowledge about managing frailty may help them to better navigate discussions with their care providers:

“Empowering our clients, definitely, with this information so they can be self-advocates for themselves. Clients and families, right? If you're going to go see your doctor, here are the five things you can ask them about. If you're overwhelmed, if you haven't seen a physician in a long time, because we have a lot of those individuals, here's the five things that you can start by talking about with them, to focus, because there is a fear for a lot of people to go seek medical assistance and help, right? So just helping them guide those conversations with their healthcare professionals.” *(Participant 1, Focus Group 3, Community Health Services Manager).*

The same participant further noted how informing patients about methods for preventing frailty would allow patients to take precautionary measures to delay and prevent frailty:

“One of the ways to capture that, is that clients are coming to us at a less disastrous period of their health journey, they're recognizing when they need that support sooner. So looking at, like, we don't capture frailty scores consistently throughout the community right now, but maybe that's something we do at what frailty level are they coming to us right now, which is probably gonna be the high, you know, totally dependent clients coming up to us. Versus, are they proactively reaching out or are they proactively seeking that support from whoever they're connected within the healthcare world? Their physician, their nurses, whatever, and are they self-identifying themselves? Or are we as providers and clinicians also self-identifying them soon enough to get those services activated in the community to prevent them from deteriorating.” *(Participant 1, Focus Group 3, Community Health Services Manager)*

A respondent in the post-module survey noted that care providers may be able to educate participants to make informed lifestyle decisions which are conducive to health and well-being, stating that *“Participating in the AVOID-based educational module can benefit clients by increasing their knowledge and empowering them to make informed lifestyle choices that promote overall health and well-being.”* Overall, participants highlighted the benefits of their increased ability to educate clients on frailty, as well as noting the importance of early identification of frailty and the advantages of taking a preventative approach to managing frailty.

### 3.7 Overarching perspectives on the AVOID educational module

Participants who completed the module were asked to provide feedback on their overall impressions of the module and share their opinions on areas for improvement through surveys and focus groups. Participants appreciated the clarity of the module:

“Clear, concise. It was short. You know, it was focused, yet it was very general … but yeah, clear, concise, short, right? And I think, because that's one thing here … depending on what team I'm dealing with … my medical outpatient unit like there's no time for education.” *(Participant 2, Focus Group 3, Team lead for home support).*

A focus group participant discussed how completing the pre- and post- module surveys enabled them to reflect on and understand their learning:

“I thought that the structure of having a pre and post-test survey, totally unique. I've never done an online session like that before so that stood out to me. As well, kind of the format, there was no, you know, read the material, do the test at the end. It was more of a … information before and after, and I thought that was unique and positive.” *(Participant 1, Focus Group 2, Occupational therapist).*

A prominent theme throughout the survey responses and focus groups was that participants felt the module could have benefited from more visual and interactive components. For example, a participant who completed the post-module survey wrote: *“The overall learning experience was great. Though learning/educational videos can also be included for a better learning experience.”* This idea was further developed by a focus group participant, who believed that increasing the amount of audio and video as well as interactive components could help make the module more engaging:

“Adding audio and video would add to it in all likelihood. I feel like it would give a little bit more impact for the course, and it would be a little bit more interactive in that way…. [A]t least then people are more engaged in it, like I don't mind reading, but I think a lot of people would be sort of like, oh, I have to read this I'm gonna read that now. You know, it's good information, but I think it can be illustrated a little better with, at least audio if not video as well.” *(Participant 2, Focus Group 2, Occupational therapist).*

Numerous survey participants expressed similar sentiments. One participant wrote “*one feedback would be to add more pictures as some people are more visual learner.”* Another survey participant wrote *“I think there should maybe be a video module explaining this as well as I learn best from those,”* while another stated, *“The module can use some images/videos for the learner to have improved experience and understanding.”*

One participant who completed the post-module survey appreciated some aspects of the AVOID framework, but felt the framework overlooked some important considerations, noting “*It addresses basic physical/social needs of humans, particularly as their health declines. Missing other aspects of humanness such as cultural, spiritual, emotional and mental wellbeing.”*

Overall, when asked to provide feedback on the AVOID Frailty educational module, participants appreciated the length, content, and opportunities to complete pre- and post- module surveys. Participants discussed concerns about the lack of interactive components and audio and visual presentation of the information in the module.

## 4 Discussion

Throughout the evaluation of the AVOID Frailty educational module, numerous objectives related to the reach of the educational module were assessed, including staff learnings from the module, perceived benefits to patients which may result from formal care providers completing the module, and recommendations for improvement of the module. Overall, the module was completed by a wide range of health care professionals and care providers. Within the first three months the module was implemented, pre-module surveys were completed by 1,509 participants, and more participants completed the module after data collection from the pre- and post- module surveys was completed.

The variety of methods by which people learned about the AVOID framework may have contributed to its reach; most participants reported learning about the framework through their employer, but participants also reported learning about the framework through other sources including educational institutions and peers. Participants described how they were able to leverage existing meetings, partnerships with different administrators, and a health authority initiative focusing on frailty to promote the module. Exposure to the AVOID Frailty educational module at meetings may have increased participants’ motivation to learn more about the AVOID framework and complete the module. This idea is supported by prior research; for example, Ditta et al. (18) found that exposing participants to videos on topics they were previously not motivated to learn about resulted in increased motivation to learn more about those topics. In sum, the AVOID Frailty educational module was completed by a wide variety of professionals, who learned about the module through a variety of existing channels that are embedded into their regular practice, which may have contributed to the uptake of the module.

In the post-module survey, participants reported increases in their knowledge of frailty prevention, assessment, and mitigation compared to the pre-module survey. Across all three domains of frailty management (prevention, assessment, and mitigation), the proportion of participants reporting having either a “Very Good” or “Excellent” understanding increased. Further, across all three domains of frailty management, the proportion of participants reporting having a “Poor” or “Very Poor” understanding decreased. This suggests that the module was effective at increasing understanding of frailty management across a breadth of prior knowledge of frailty management. Participants who previously had an average understanding of frailty management had the opportunity to progress to a very good or excellent understanding of frailty management, while participants who previously had a poor understanding of frailty management may have progressed to an average understanding of frailty management. Indeed, a strength of the module is its effectiveness for increasing self-reported knowledge of frailty management for people with wide ranges of prior knowledge, rather than benefiting only people with a specific level of prior understanding of frailty management.

Participants in focus groups and surveys frequently expressed interest in using resources from the AVOID Frailty educational module including the PRCHA website, the Comprehensive Geriatric Assessment (12), and the Clinical Frailty Scale (11). This willingness of participants to access resources related to healthy aging is consistent with the goals of PRCHA, which is a network promoting healthy aging by working with the public, the health care community, non-profit organizations, and academics. The AVOID Frailty educational module is promoted on the PRCHA website, and in turn the AVOID Frailty educational module provides access to external resources related to frailty management. Access to resources can help people with complex care needs achieve their health goals, but community resources remain underused, and lack of awareness is a barrier to people’s ability to use these resources (19). The Digital Divide is a widely discussed phenomenon which refers to disparities in access to information and communications technologies between demographics, which further contributes to inequalities in accessing health care information (20). Yu and Meng (21) found that internet access can reduce barriers to health care access associated with income inequality. Enabling access to internet resources for managing frailty may help mitigate inequalities to health care access associated with income disparities for older adults. It is also important to ensure older adults, public members, and care providers who can navigate these online resources can access simple functions that allow for information printing, so they can bring online information to those who might not have internet access.

Participants in focus groups and surveys discussed challenges related to incorporating information from the module into their clinical practice. These challenges included a lack of time and resources for promoting frailty prevention, a care model which is focused on frailty mitigation rather than prevention, and an apparent lack of tangible steps which could help them incorporate learnings from the module into their practice. The overreliance on treatment compared to prevention of chronic conditions in Canada and the United States has been widely discussed (22, 23). Frailty prevention interventions have demonstrated efficacy in preventing pre-frail persons from becoming frail and in reducing associated health costs (24). Given the increased economic costs associated with caring for frail compared to non-frail older adults (2, 25), increasing awareness and initiatives for frailty prevention in Canada may be important for improving quality of life for older adults as well as saving on associated health care costs.

Many participants showed interest in incorporating proactive approaches to preventing frailty but noted that limitations in resources may impede care models from adopting frailty prevention strategies. Even with substantial interest in system-wide changes to models of care to focus on frailty prevention rather than treatment, without sufficient staff resources these changes may not be feasible. Thus, a prudent approach may be to focus on recruitment and retention of staff while simultaneously attempting to restructure models of care. A multi-pronged approach to staff recruitment and retention may be necessary for restructuring frailty care in Canada to be preventative rather than responsive. Firstly, sufficient staff such as clinical nurse educators and policymakers to design policy around frailty prevention will need to be employed. In addition, it will be necessary to recruit sufficient frontline workers to allow appropriate frontline worker time to be dedicated to frailty prevention training.

Difficulty understanding how to use the information from the AVOID Frailty educational module to inform clinical practice was another challenge discussed by participants. It may be that part of this challenge stems from the module being designed for a broad range of health care professionals, so it was not intended to provide specific guidance for clinicians on how to manage frailty in their daily work. Still, this may represent a disconnect between module developers and clinical end-users in understanding of the purpose of the module: during development, module developers focused on high-level information about the management of frailty, while some clinical end-users expected information about how to guide their practice. Multiple solutions may be effective for bridging this disconnect. Firstly, module developers may wish to clarify throughout the module that it is intended to provide a high-level overview of frailty management, rather than specific clinical guidance, to guide end-user expectations about the content of the module. Secondly, module developers may choose to design additional modules or components of the AVOID Frailty educational module which do discuss profession-specific strategies for managing frailty. Integrating end-user perspectives throughout early stages of module development may be an effective strategy for ensuring that learning goals are clearly identified throughout the development process.

Two themes which emerged through discussions about benefits to patients which could occur from staff participation in the AVOID Frailty educational module were the potential for patients to be increasingly empowered in their discussions with formal care providers, and the ability for patients to take proactive measures against frailty. When formal care providers are educated in the AVOID framework, they may better educate their patients about the AVOID framework, and structure discussions with patients around the AVOID framework. Further, if patients become aware of the AVOID framework through a health care provider, they may be better equipped for effective discussions and advocacy with their health care providers. For instance, if patients and formal care providers alike understand that activity, vaccination, optimization of medications, interaction, and diet are factors affecting frailty, they can effectively work together to create a structured plan for preventing or reversing frailty, since patients and care providers will be aligned in their understanding of the necessary factors for preventing or reversing frailty.

If individuals understand the factors affecting frailty, they may be able to take proactive measures to prevent or reverse frailty. Frailty can be prevented or reversed through lifestyle changes, such as increasing physical activity, improving diet, and increasing social interaction (8, 14). Importantly, this provides a way for preventative measures to be taken against frailty without substantially increasing the burden on formal care providers. Formal care providers may be unable to spend time developing individualized, tailored frailty prevention plans with each of their patients. However, if patients are aware of the factors affecting frailty, they may be able to develop plans themselves, which may improve their self-efficacy as well as help prevent or reverse frailty through improved intrinsic capacity. Self-efficacy, one’s belief that they will be able to act in ways which produce desired outcomes in given situations, may be increased through interventions which promote self-care (26). In turn, self-efficacy is associated with behaviors which can promote healthy aging in older adults (27). Empowering patients with the knowledge to prevent frailty themselves may serve a dual purpose of preventing or reversing frailty as well as improving self-efficacy. Notably, these potential benefits are contingent on formal care providers educating their patients. Indeed, the necessity for health care providers to educate patients has been widely discussed (26, 28). Having formal care providers introduce the AVOID framework to patients has the potential to produce benefits including promoting structured discussion with their formal care providers, enabling them to take proactive measures for managing frailty, and increasing self-efficacy.

Formal care providers who participated in surveys and focus groups appreciated the length of the module, which generally took about 20 min to complete. Keeping eLearning educational modules concise may be important for maintaining high completion rates. For instance, Pomales-García & Liu (29) administered web-based educational modules of different lengths and found that as the length of the module increased from seven to 20 min, participants were less likely to want to finish the module, and more likely to want to either pause the module partway through and complete it later or not complete the module. Further, participants rated longer modules as less exciting (29).

Formal care provider participants suggested that the module could have been improved with the addition of more interactive material and more audio and video components. Indeed, it has been found that including interactive activities throughout the learning process can improve learning outcomes (30). Some research has suggested that video learning opportunities are important for promoting eLearning acceptance (31). However, it is worth considering that participants who complete eLearning modules with video and audio components may perceive the modules to take more time to complete than text-only modules of the same length (29). Zhang et al. (32) found that online learning environments with interactive videos facilitated learning performance compared to online learning environments with non-interactive videos or no videos. A lack of interactivity is a common design flaw in evidence-based practice instruction modules (33). Interactive components and audio and video elements are important for sustaining engagement and interest in online educational modules.

### 4.1 Recommendations for module developers

**Figure 5:**
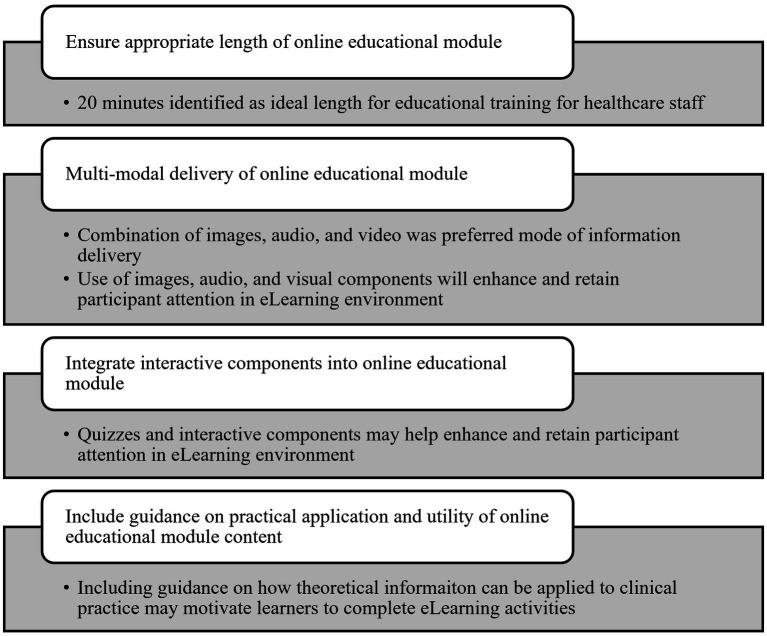
Recommendations for eLearning module developers.

Based on this evaluation of the AVOID Frailty educational module and as shown in Figure 5, four key recommendations are proposed for developers of eLearning educational modules:

1. Ensure educational modules are an appropriate length. In our evaluation, 20 min was identified as an ideal length of time for educational modules by healthcare staff.
2. Deliver online educational modules in multi-modal format. Healthcare staff in our evaluation noted that combining images, as well as audio and video components, was a preferred method of information delivery and may help enhance and retain participant attention.
3. Integrate interactive components into online educational modules. Quizzes and interactive components were identified by participants in our evaluation as beneficial for improving the experience of completing the AVOID Frailty educational module.
4. Include guidance on practical application and utility of online educational module content; in particular, discuss how theoretical information can be used to inform hands-on practice.

### 4.2 Strengths and limitations

A strength of this study was the breadth of feedback obtained from conducting surveys and focus groups with diverse health care professionals, which allowed collection of multiple perspectives on the module. Additionally, the close working relationship between an interdisciplinary evaluation team involving health care leaders, clinical staff, academics, and trainees was integral to the co-creation of this evaluation. Partnerships emerged throughout the development and promotion of the module, such as the module being shared with smaller community organizations through the networks of larger non-profit organizations. Since its initial implementation, the module has been shared with other regional health authorities, which has resulted in greater awareness of the module and a greater number of health care staff completing the module. Functionality issues with the module which were identified throughout this evaluation were subsequently addressed and resolved by module developers and web developers.

A limitation of our study is the different sample profiles between the focus groups and surveys. Sixty-one percent of participants who completed the survey identified as community members, unregulated health professionals (e.g., community health workers and health care aides), or other participants. In our focus groups, only one of four focus groups was held with community health workers and health care aides. As a result, our insights from focus groups may be disproportionally reflective of the views of allied health professionals, nurses, and health care managers, when these staff comprised a minority of participants who completed the module.

### 4.3 Future opportunities

Notably, the improvements in knowledge of frailty assessment, mitigation, and prevention reported in this evaluation were based on self-reported measures from participants. Future evaluation may wish to use other methods for evaluating changes in knowledge resulting from completion of the AVOID Frailty educational module, such as pre- and post- module quizzes which ask questions to probe participants’ knowledge of frailty management.

Warren et al. (7) also suggested longitudinal evaluation on care providers’ practice change and patient outcomes as result of frailty education. It may be beneficial to conduct a follow-up study with a group of participants who completed the AVOID Frailty educational module to investigate the long-term impacts of the module on their practice. Holding follow-up focus groups with participants who completed the module would allow further understanding of which elements of the module were best retained, and the long-term ways in which the module informed clinicians’ practice.

Another opportunity for future evaluation would be to solicit perspectives from a broader range of staff who will complete the module. In British Columbia, PRCHA plans to ultimately offer the module in multiple health authorities throughout the province. Each health authority in British Columbia serves a region with different needs, such as rural areas which can face unique challenges including limited internet access and difficulties recruiting and retaining sufficient personnel (34, 35).

## 5 Conclusion

Overall, the AVOID Frailty educational module was effective for increasing formal care providers’ self-reported understanding of frailty assessment, mitigation, and prevention. Importantly, most participants indicated that they intended to use resources from the module, underscoring the relevance of resources included throughout the module. Participants discussed the feasibility of incorporating strategies from the module into clinical practice, noting that while the module was informative, it lacked information about how to incorporate the theoretical information which was provided into clinical practice. Further, participants discussed potential benefits to clients which may result from formal care provider participation in the module, including the potential for clinicians and patients to refer to the AVOID framework to facilitate discussions around frailty. It is recommended that developers of eLearning educational modules consider the length of modules (20 min may be an effective length), include audio and video components, and incorporate interactive components to maximize engagement with educational modules. Additionally, eLearning module developers should strive to either include information about practical utility of resources in the module or clarify that the module is intended to provide theoretical information to maximize module engagement and education impact. Future investigations should employ objective measures to quantify participant learnings resulting from the AVOID Frailty educational module, such as pre- and post- module quizzes to assess the impact of the module. Further, it may be useful to examine long-term impacts of the effects of the educational module on clinicians’ practice.

## Data Availability

Data from this quality improvement initiative are not publicly available but may be requested from the corresponding author.
