# Supplementary material for: Sustainable implementation of a frailty education program for formal health care providers
Source: Front Public Health. 2025 Nov 12;13:1654098. doi: 10.3389/fpubh.2025.1654098 (PMC12646874; doi:10.3389/fpubh.2025.1654098)
Supplement: Supplementary file 1 [file Table_1.DOCX]

Supplementary Material

# 1. Survey Questions

**1.1 Pre-survey**

Completion requirements

**Done:** Submit feedback

Top of Form

Continue

Bottom of Form

Top of Form

Mode: Anonymous

1. What is your Profession?

 Not selected
 Nurse
 Nurse Practitioner
 Allied Health Professional
 Administrator
 Academic/Researcher
 Leadership/Corporate
 Community Member
 Ethicist
 Physician
 Unregulated Health Professional
 Student
 Other

2. On a scale of 1 (Very Poor) to 7 (Excellent), please rate your understanding of frailty assessment (i.e. assessing the level of frailty of a client).

 Not selected  (7) Excellent  (6) Very Good  (5) Above Average  (4) Average  (3) Below Average  (2) Poor  (1) Very Poor

3. On a scale of 1 (Very Poor) to 7 (Excellent), please rate your understanding of frailty mitigation (i.e. minimizing the impact of a client’s frailty).

 Not selected  (7) Excellent  (6) Very Good  (5) Above Average  (4) Average  (3) Below Average  (2) Poor  (1) Very Poor

4. On a scale of 1 (Very Poor) to 7 (Excellent), please rate your understanding of frailty prevention (i.e. identifying and managing risk factors that may contribute to the progression of a client’s frailty).

 Not selected  (7) Excellent  (6) Very Good  (5) Above Average  (4) Average  (3) Below Average  (2) Poor  (1) Very Poor

5. Have you previously heard about the AVOID Frailty Framework?

 Not selected  Yes  No

6. If yes, where did you hear about the AVOID Frailty Framework? (Skip if answer to Q5 was “No”)

 Not selected
 Canadian Frailty Network website
 Employer
 Peer
 Educational Institution
 Other

7. What are your learning goals? Is there anything specific you hope to learn about frailty through completion of this module?

**1.2 Post-survey**

Bottom of Form

Top of Form

Mode: Anonymous

1. On a scale of 1 (Very Poor) to 7 (Excellent), please rate your understanding of frailty assessment (i.e. assessing the level of frailty of a client).

 Not selected  (7) Excellent  (6) Very Good  (5) Above Average  (4) Average  (3) Below Average  (2) Poor  (1) Very Poor

2. On a scale of 1 (Very Poor) to 7 (Excellent), please rate your understanding of frailty mitigation (i.e. minimizing the impact of a client’s frailty).

 Not selected  (7) Excellent  (6) Very Good  (5) Above Average  (4) Average  (3) Below Average  (2) Poor  (1) Very Poor

3. On a scale of 1 (Very Poor) to 7 (Excellent), please rate your understanding of frailty prevention (i.e. identifying and managing risk factors that may contribute to the progression of a client’s frailty).

 Not selected  (7) Excellent  (6) Very Good  (5) Above Average  (4) Average  (3) Below Average  (2) Poor  (1) Very Poor

4. How has your knowledge of frailty assessment changed after completion of the educational module?

 Not selected
 No change in knowledge
 Confirmed my existing knowledge only
 Provided me with some new knowledge AND confirmed my existing knowledge
 Provided me with a substantial amount of new knowledge AND confirmed my existing knowledge
 Provided me with some new knowledge and DID NOT confirm my existing knowledge
 Provided me with a substantial amount of new knowledge and DID NOT confirm my existing knowledge

5. How has your knowledge of frailty mitigation changed after completion of the educational module?

 Not selected
 No change in knowledge
 Confirmed my existing knowledge only
 Provided me with some new knowledge AND confirmed my existing knowledge
 Provided me with a substantial amount of new knowledge AND confirmed my existing knowledge
 Provided me with some new knowledge and DID NOT confirm my existing knowledge
 Provided me with a substantial amount of new knowledge and DID NOT confirm my existing knowledge

6. How has your knowledge of frailty prevention changed after completion of the educational module?

 Not selected
 No change in knowledge
 Confirmed my existing knowledge only
 Provided me with some new knowledge AND confirmed my existing knowledge
 Provided me with a substantial amount of new knowledge AND confirmed my existing knowledge
 Provided me with some new knowledge and DID NOT confirm my existing knowledge
 Provided me with a substantial amount of new knowledge and DID NOT confirm my existing knowledge

7. What are two of your key takeaways from the AVOID-based educational module?

8. Will you be utilizing the resources from this module?

 Not selected  Yes  No

9. The AVOID education workshop referenced a number of external resources associated with healthy aging and frailty management. What resources, if any, did you find most useful?

10. Did you find this educational module helpful?

 Not selected  Yes  No

11. What potential benefits do you perceive for your clients/patients as a result of your participation in the AVOID-based educational module?

12. What were your learning goals, and were they met? Please describe.

13. Would you recommend this educational module to a friend/co-worker?

 Not selected  Yes  No

14. Please provide your feedback/ideas on how the overall learning experience can be improved.

**2. Focus Groups Scripts**

**2.1 Managers and Leaders Perspectives on PRCHA AVOID Educational Module** **Semi-Structured Focus Group Script**

**These questions are designed to get a sense of participants’ backgrounds.**

1. Please tell me a bit about yourself and describe your position and role.
   1. How long have you been involved in your current position?
   2. How did you learn about the AVOID Frailty educational module?

**These questions are designed to explore participants’ perspectives of the module and attitudes towards incorporating content from the module into their practice.**

1. What are your overall thoughts towards the AVOID Frailty educational module?
   1. What did you find most helpful about the educational module?
   2. Was there any content in the educational module that was redundant or not helpful?
   3. Was there any content missing from the educational module which should have been included?
   4. How could the educational module be improved?
2. How feasible will it be to incorporate methods from this module such as the AVOID/frailty assessments into your practice?
   1. What barriers may pose a challenge to successful implementation?

- Probe for comments about current limited adoption of AVOID education, potential concerns about ability to complete newly learned frailty assessments.
  1. How could these barriers be addressed?
- Probe for comments on increasing numbers of in-person workshops on assessing clients for frailty, etc.

1. Are there any strategies or methods from the module that you have already begun to implement into your practice that you could share about?
2. Which methods or ideas from the module are you particularly excited about implementing into practice?
3. Are there methods or ideas from the module you do not intend to implement into practice?

- Probe participants for explanations why they intend not to incorporate methods from the module.

**These questions are designed to explore participants’ ideas on facilitating staff participation and promoting widespread adoption of AVOID-based strategies for addressing frailty.**

1. How could employees be motivated to complete the AVOID module?

- Probe for comments about incentivizing staff, raising awareness of module.

1. What strategies could support the staff in implementing knowledge from the module into their practice?
2. How can this course be aligned with existing training programs and workflows to ensure integration into healthcare operations?
3. What are the benefits of the implementation of the AVOID frailty framework that directly relate to clinical practice and patient care?
4. How can the impact of this course on clinical practice be measured?

- Probe for impact on patient outcomes and the overall quality of care

1. Is there anything else I haven’t asked that you’d like to share?

**2.2 Nurse and Allied Health Professional Perspectives on PRCHA AVOID Educational Module Semi-Structured Focus Group Script**

**These questions are designed to get a sense of participants’ backgrounds and previous knowledge about frailty.**

1. Please tell me a bit about yourself and describe your position and role.
   1. How long have you been involved in your current position?
   2. How did you learn about the AVOID Frailty educational module?
2. How would you describe your knowledge of frailty before completing this module?
   1. How did you define frailty?
   2. How did you assess and prevent frailty?

**These questions are designed to probe changes in their knowledge of frailty after completion of the module and perceived benefits to completing the training.**

1. How has your understanding of frailty assessment changed after completing this educational module?

- Probe for comments about
- important lessons learned,
- new strategies for assessing frailty.

1. How has your understanding of frailty prevention changed after completing this educational module?

- Probe for comments about
- important lessons learned,
- new strategies for preventing frailty.

1. How has your understanding of frailty mitigation changed after completing this educational module?

- Probe for comments about
- important lessons learned,
- new strategies for mitigating frailty.

1. What information did you find most valuable from the module?

- Probe for new learning.

1. How might your interest in this module affect the provision of patient care?

**These questions are designed to explore participants’ perspectives of the module and attitudes towards incorporating content from the module into their practice.**

1. How feasible will it be to incorporate methods from this module, such as the AVOID/frailty assessments, into your practice?
   1. What barriers may pose a challenge to successful implementation?

- Probe for comments about current limited adoption of AVOID education, potential concerns about ability to complete newly learned frailty assessments.
  1. How could these barriers be addressed?
- Probe for comments on increasing numbers of in-person workshops on assessing clients for frailty, etc.

1. Are there any strategies or methods from the module that you have already begun to implement into your practice that you could share about?
2. Which methods or ideas from the module are you particularly excited about implementing into your practice?
3. Are there methods or ideas from the module you do not intend to implement into practice?

- Probe participants for explanations why they intend not to incorporate methods from the module.

1. What are your overall thoughts towards the AVOID Frailty educational module?
   1. What did you find most helpful about the educational module?
   2. Was there any content in the educational module that was redundant or not helpful?
   3. Was there any content missing from the educational module which should have been included?
   4. How could the educational module be improved?
2. Is there anything else I haven’t asked that you’d like to share?

**2.3 Community Health Workers’ Perspectives on PRCHA AVOID Educational Module Semi-Structured Focus Group Script**

**These questions are designed to get a sense of participants’ backgrounds and previous knowledge about frailty.**

1. Please tell me a bit about yourself and describe your position and role.
2. How long have you been involved in your current position?
3. How did you learn about the AVOID Frailty educational module?
4. How would you describe your knowledge of frailty before completing this module?
5. How did you define frailty?
6. How did you assess and prevent frailty?

**These questions are designed to probe changes in their knowledge of frailty after completion of the module and perceived benefits to completing the training.**

1. How has your understanding of frailty assessment changed after completing this educational module?

- Probe for comments about
- important lessons learned,
- new strategies for assessing frailty.

1. How has your understanding of frailty prevention changed after completing this educational module?

- Probe for comments about
- important lessons learned,
- new strategies for preventing frailty.

1. How has your understanding of frailty mitigation changed after completing this educational module?

- Probe for comments about
- important lessons learned,
- new strategies for mitigating frailty.

1. What information did you find most valuable from the module?

- Probe for new learning.

1. How might your interest in this module affect the provision of patient care?
2. Are there any strategies or methods from the module that you have already begun to implement into your practice that you could share about?
3. Which methods or ideas from the module are you particularly excited about implementing into your practice?
4. Are there methods or ideas from the module which you do not intend to implement into practice?

- Probe participants for explanations why they intend not to incorporate methods from the module.

1. What are your overall thoughts towards the AVOID Frailty educational module?
   1. What did you find most helpful about the educational module?
   2. Was there any content in the educational module that was redundant or not helpful?
   3. Was there any content missing from the educational module which should have been included?
   4. How could the educational module be improved?
2. Is there anything else I haven’t asked that you’d like to share?
